# Left Ventricle Segmentation in Cardiac MR Images via an Improved ResUnet

**DOI:** 10.1155/2022/8669305

**Published:** 2022-07-08

**Authors:** Shengzhou Xu, Haoran Lu, Shiyu Cheng, Chengdan Pei

**Affiliations:** ^1^College of Computer Science, South-Central Minzu University, Wuhan 430074, China; ^2^Network Information Center, Wuhan Institute of Technology, Wuhan 430205, China

## Abstract

Cardiovascular diseases are reported as the leading cause of death around the world. Automatic segmentation of the left ventricle (LV) from magnetic resonance (MR) images is essential for an early diagnosis. An enhanced ResUnet is proposed in this paper to improve the performance of extracting LV endocardium and epicardium from MR images, improving the accuracy of the model by introducing a medium skip connection for the contracting path and a short skip connection for the residual unit. Also, a depth-wise separable convolution replaces the typical convolution operation to improve training efficiency. In the MICCAI 2009 LV segmentation challenge test dataset, the percentages of “good” contours, dice metric, and average perpendicular distance of endocardium (epicardium) are 99.12% ± 2.29%(100% ± 0%), 0.93 ± 0.02 (0.96 ± 0.01), and 1.60 ± 0.42 mm (1.37 ± 0.23 mm), respectively. Experimental results demonstrate that the proposed model obtains promising performance and outperforms state-of-the-art methods. By incorporating these various skip connections, the segmentation accuracy of the model is significantly improved, while the depth-wise separable convolution also improves the model efficiency.

## 1. Introduction

Cardiovascular diseases are the leading cause of death worldwide [1]. With the rapid development of medical imaging technology, high-resolution images for noninvasive assessments of the function and structure of the cardiovascular system can be provided by magnetic resonance (MR) [2]. Segmentation of the left ventricular (LV) endocardium and epicardium from MR images is crucial for cardiologists to evaluate LV functional parameters quantitatively. However, the automatic segmentation of LV remains challenging due to the interference of noise, causing feature boundaries to be blurred and the introduction of outflow tract problems in some MR images [3]. Many models and methods have been proposed [4–6] that can be categorized as traditional segmentation methods [7, 8], deep learning-based methods, or a combination of each. Traditional methods [9, 10] typically require manual design and extraction of features that represent the target. These approaches also suffer from low accuracy and limited robustness.

Recently, convolutional neural networks (CNN), such as LeNet [11], AlexNet [12], and GoogleNet [13], were introduced to solve image classification problems. Some CNN models solve the problem of image segmentation by obtaining the classification information of each pixel to achieve a pixel-level classification [14, 15]. U-Net is an architecture based on a fully convolutional neural network (FCN) proposed for biomedical image segmentation [16].

Many researchers have demonstrated good progress leveraging CNN models, including U-Net, to segment LV from MR images [17, 18]. Abdeltawab et al. achieved LV segmentation with U-Net featuring a loss function composed of binary cross-entropy and the sum of the sensitivity and specificity [19]. This technique was performed with good segmentation accuracy on LV segmentation without improvements to the U-Net model structure, and opportunities exist for enhancements. Yuan et al. implemented a multiscale fusion learning framework and obtained better LV segmentation accuracy based on CNN regression [20]. However, this approach also has room for improvement in utilizing the original image features. Tao et al. developed a fully automated cine MRI analysis system with moderate results in LV segmentation [21]. The changes introduced to the network model in this work are insufficient, leaving opportunities for improving segmentation accuracy. Moradi et al. concatenated the feature maps of each level of the U-Net decoding path and achieved ideal results in echocardiography [22]. However, this method does not strengthen the encoding ability of the model. Kerfoot et al. proposed the residual U-Net (ResUnet), which significantly improved the performance of the original U-Net by introducing residual blocks [23] in the convolution process of downsampling and upsampling. However, this enhancement does not reuse enough features of the original graph and increases the complexity of the network. To alleviate excessive time and memory consumption during training, Han et al. proposed a ghost module that reduces the time and memory of convolution network training by a depth-wise separable convolution [24]. By combining a multiscale segmentation network and a co-discrimination network, Wu et al. proposed a GAN model for LV segmentation that guarantees the ground truth and unlabeled samples are trained in the segmentation network [25]. However, the training efficiency and stability of GAN networks have always been a troublesome issue. Avendi et al. developed a fully automatic LV segmentation tool by combining a deep learning model with a deformable model [26]. Although these methods have demonstrated good performance in left ventricular segmentations, some gaps remain in the accuracy and efficiency required for clinical application. The accurate endocardium and epicardium segmentation plays a crucial role in the calculation of ejection fraction (EF) and left ventricular mass (LVM), which are important indicators for evaluating whether the heart is healthy.

In this paper, we propose an enhanced ResUnet for improving the accuracy and efficiency of the endocardium and epicardium extracting process from cardiac MR images. The designed architecture is based on ResUnet with the layers of the contracting and expanding paths defined using residual units, making full use of the features in each layer, especially the original image features. Specifically, in addition to the long skip connections between the contracting and expanding paths, medium skip connection is introduced for the contracting path. In addition, a short skip connection is introduced for the residual unit to improve the sensitivity of the model to gradient changes and the recoverability of spatial information lost during downsampling. Also, a depth-wise separable convolution is incorporated to replace the typical convolution operation to improve training efficiency. The proposed model is evaluated on the MICCAI 2009 and LVQuan18 datasets, with results suggesting the effectiveness and advantages provided by the implementation of our proposed model.

## 2. Materials and Methods

### 2.1. Datasets and Evaluation

Our experiments were performed on the cardiac short-axis cine MR images provided by the MICCAI 2009 [27] and LVQuan18 [28] challenge datasets. Forty-five MR cases are included in MICCAI 2009, with four cases of heart failure with ischemia (HF-I), four cases of heart failure without ischemia (HF-NI), four hypertrophy (HYP) cases, and three cases of normal (N). These datasets are divided into training, validation, and testing sets, each containing an average of 15 cases. For each case, approximately six to 12 short-axis cine images with a specific dimension of 256 × 256 pixels and thickness of 8–10 mm are obtained from the atrioventricular ring to the apex. The endocardium of all slices in the end-diastole (ED) and end-systole (ES) cardiac phases is drawn and confirmed by two cardiologists, and the epicardium of all slices is drawn only in the ED cardiac phase. These manual annotations provide the ground truth of the segmentation for evaluation purposes.

To verify the effectiveness and adaptability of our model, we also evaluated with another updated dataset, LVQuan18, consisting of 145 cases, each with 20 frames. The ROIs of the 80 × 80 pixels in the LVQuan18 set originate from three hospitals affiliated with two healthcare centers, St. Joseph's Healthcare and London Healthcare Center. For each frame, the ground truth of the segmentation is provided for evaluation purposes.

We evaluate the segmentation performance with three measures in the MICCAI 2009 challenge set, including the percentage of “good” contours (PGC), the average perpendicular distance (APD), and the dice metric (DM) of the “good” contours. APD measures the similarity between the automatically segmented contour and the corresponding ground truth by calculating the average distance between all contour points [25]. A segmentation is classified as “good” if the APD is less than 5 mm [26]. Only the slices with good segmentation participate in the calculation of DM, which is defined as the similarity between the area of the auto-segmented contour *A*_s_ and the area of the ground truth *A*_g_, expressed as

(1) $$\begin{array}{c} \text{DM}=2\frac{\left(A_{s}\cap A_{\text{g}}\right)}{\left(A_{s}+A_{g}\right)}. \end{array}$$

The resulting DM is between 0 and 1, such that a greater DM value corresponds to the automated segmentation result being closer to the ground truth.

The EF and LVM are important indexes to evaluate if the heart is healthy. These two measures are calculated based on the autosegmentation results, defined as

(2) $$\begin{array}{c} \text{LVM}=\left(V_{\text{epi}}^{\text{ED}}-V_{\text{end}}^{\text{ED}}\right)*1.05, \end{array}$$

(3) $$\begin{array}{c} \text{EF}=\frac{\left(V_{\text{end}}^{\text{ED}}-V_{\text{end}}^{\text{ES}}\right)}{V_{\text{end}}^{\text{ED}}}*100\%, \end{array}$$

where *V*_epi_^ED^ and *V*_end_^ED^ are the epicardial and endocardial volumes in the ED phase, respectively, and *V*_end_^ES^ is the endocardial volume in the ES phase.

### 2.2. Method

**Figure 1:**
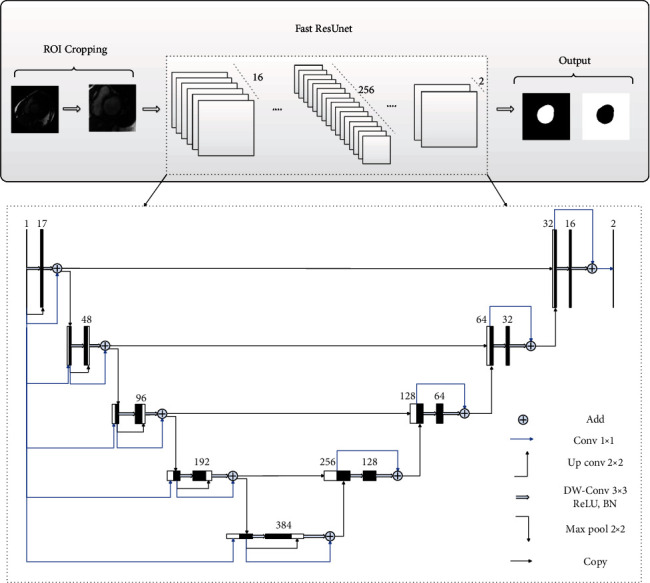
The proposed segmentation model with labels Add (addition), Conv (convolution), DW-Conv (depth-wise convolution), BN (batch normalization), and ReLU (rectified linear unit).

**Figure 2:**
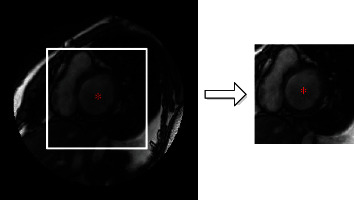
ROI cropping. The image on the left is the original slice, and the white box represents the ROI containing the LV. The image on the right is the corresponding cropped ROIs.

**Figure 3:**
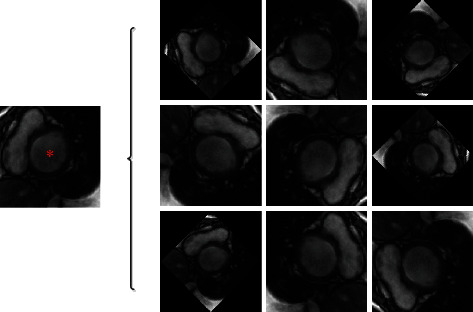
Data augmentation. The image on the left is the corresponding cropped ROI, and those on the right are augmented ROIs.

The proposed segmentation method is shown in Figure 1. To reduce memory consumption and computing time, the ROIs with a size of 128 × 128 pixels are extracted (as illustrated in Figure 2) by the ROI cropping method based on k-means clustering and a threshold adjustment [29]. Due to the limited number of images available in the MICCAI 2009 dataset, data augmentation is applied to improve the training effect of the model. As shown in Figure 3, the ROIs are rotated 45, 90, 135, 180, 225, 270, and 315 degrees clockwise, then flipped horizontally and vertically. This augmentation increases the number of images in the training dataset to ten times the original quantity.

These cropped ROIs are input to the improved ResUnet model, as presented in Figure 1. The model comprises contracting and symmetric expanding paths, which are defined using residual units. Compared with the residual U-Net, our model is enhanced through two features. First, various types of skip connections are introduced to improve the accuracy of the model, and second, a depth-wise separable convolution is introduced to improve efficiency.

Also shown in Figure 1, short, medium, and long skip connections are applied for propagating information to improve the performance of the network. Each residual unit of the contracting path consists of a residual function and two short skip connections represented by a thin blue and black arrow, respectively. These short skip connections copy features from the beginning to the subsequent layer of the current step to enhance feature reuse, enrich the feature diversity of the network, and improve the sensitivity of the network to gradient changes. For the residual function, batch normalization (BN) and a rectified linear unit (ReLU) are implemented after the convolution layers.

A medium skip connection, represented by the blue arrow in Figure 1 and starting from the input of the model, is introduced to the contracting path to enhance feature reuse and improve the representation capability of the network. For each step in the contracting path, the input of the model is concatenated with the output from the downsampling of the previous step through the medium skip connection. To match the inputs and outputs, the input of the model is processed by a 1 × 1 convolution, represented by the thin blue arrow in the figure, to adjust the number and spatial resolution of the feature maps.

Following the contracting path, a residual unit is incorporated as transition layers for the subsequent expanding path, implemented with residual units and up convolution. To recover spatial information lost during downsampling, long skip connections, represented by thin black arrows in Figure 1, connect low-level features in the contracting path with high-level features in the expanding path. At the end of the expanding path, a 1 × 1 convolution and SoftMax function, represented by the horizontal black arrow in the figure, obtain the segmentation result consistent with the spatial resolution of the input image.

**Figure 4:**
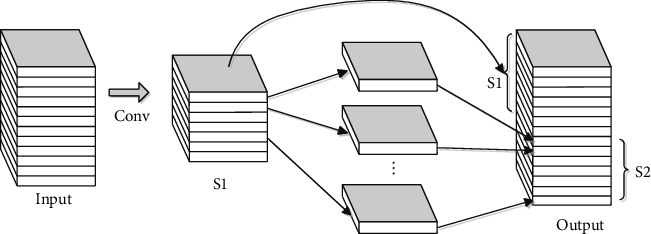
Depth-wise separable convolution.

With the introduction of these skip connections, the performance of the network is improved while increasing the time cost of the training process due to the additional parameters. Therefore, a depth-wise separable convolution (followed by a BN and ReLU) replaces the typical convolution, as shown in Figure 4, to improve the training efficiency. For example, suppose the size of the input features is *m* × *n* × *c*_1_, and the output is *m* × *n* × *c*_2_. First, the 3 × 3 convolution is applied to the input features to obtain the feature map *S*1 with a size of *m* × *n* × *c*_2_/2. Then, the separable convolution with a kernel of 3 × 3 is applied on *S*1 to obtain feature maps *S*2 with a size of *m* × *n* × *c*_2_/2. Finally, the features *S*1 and *S*2 are concatenated to form the output with a size of *m* × *n* × *c*_2_.

## 3. Experimental Results

### 3.1. Environment

Our experiments were implemented on Windows 10 using TensorFlow with cuDNN 7.0 and CUDA 9.0, equipped with an Intel® Core 2.6 GHz CPU, 32 GB of RAM, and Nvidia GeForce GTX1660 (6 GB RAM). The learning rate of the network model was dynamically and linearly adjusted from an initial value of 0.001 to 0.0001 at the 120^th^ epoch.

To facilitate comparisons with previous models, we applied the same dataset division method recommended by MICCAI 2009, as used in the relevant literature. The training and validation sets of 30 cases trained our model, and the test set of 15 cases was used for testing model performance.

### 3.2. Results

**Figure 5:**
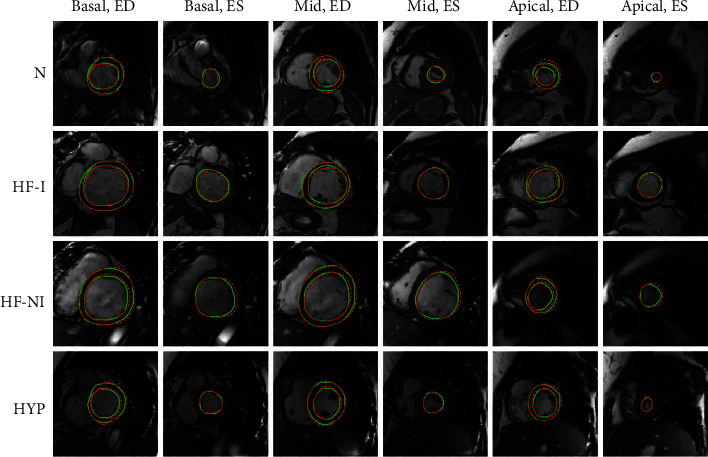
Automatic segmentation (solid green line) and ground truth (dotted red line) in the basial slice, midslice, and apical slice for four types of patients, labeled with Basal (basal slice), Mid (midslice), Apical (apical slice), ED (end-diastole), ES (end-systole), N (normal), HF-I (heart failure with ischemia), HF-NI (heart failure without ischemia), and HYP (hypertrophy).

Figure 5 illustrates the segmentation results of our proposed model (solid green line) and ground truth (dotted red line) for four types of patients. Each row, oriented from the top to bottom of the figure, corresponds to a case of N, HF-I, HF-NI, and HYP, respectively. The first and second columns are the segmentation results of the basal slice in the ED cardiac phase and ES cardiac phase, respectively. The third and fourth columns include the results for the corresponding middle slice, and the fifth and sixth columns for the apical slice. Because the epicardium contour in the ES phase is not used in the calculations of LVM and EF, the corresponding ground truth and segmentation results are omitted. For each case in Figure 5, the consistency between the segmentation results and the ground truth is very high, whether a basal slice, middle slice, or apical slice in the ED cardiac phases or ES phases.

To analyze our segmentation results quantitatively, the PGC, APD, and DM values of the segmentation results of the endocardium and epicardium for 15 test cases are listed in Table 1. Our model achieves 100% PGC for epicardium segmentation in all test cases. This result suggests that epicardium segmentation in all slices of all cases is “good” (an APD value less than 5 mm). For the endocardium segmentation, 13 of 15 cases achieve 100% PGC, and only a few slices in the SC-HYP-37 and SC-N-07 failed to achieve “good” segmentation results. The mean ± SD value of APD for the endocardium and epicardium are 1.60 ± 0.42 mm and 1.37 ± 0.23 mm, respectively, while the DM values are 0.93 ± 0.02 and 0.96 ± 0.01, respectively.

**Figure 6:**
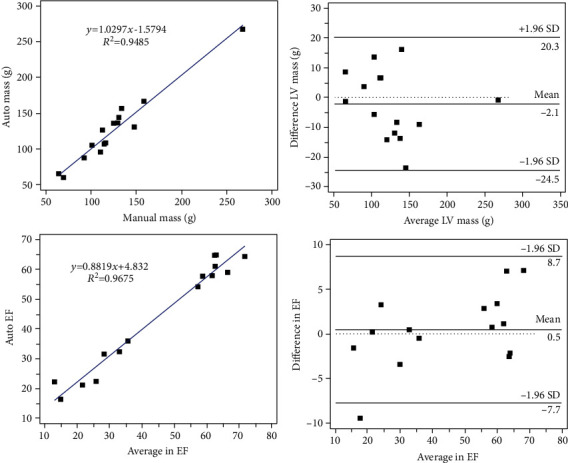
Regression and Bland-Altman plots for LVM and EF. The left side is the LVM and EF regression curves, and the right side is the Bland-Altman plots of LVM and EF.

Assessing the usefulness of our segmentation results in the two clinical indicators of the LVM and EF is considered in the regression of LVM and EF shown on the left of Figure 6. The coefficients of determination R2 for the LVM and EF are close to 0.95 and 0.97, respectively, and the slopes are approximately 1.03 and 0.89, respectively. The Bland-Altman analysis results are plotted on the right of Figure 6. For the difference between the automatic and manual LVM/EF results, the mean ± 1.96 SD value is −2.1 g ± 22.4 g/0.5 ± 7.2, the *p* values of mean bias is 0.1518/0.5291, and the confidence interval is (-24.5 g, 20.3 g)/(-7.7, 8.7), respectively. The number of cases outside the confidence intervals for the LVM is 0/15 (0.0%) and 1/15 (6.7%) for EF. The regression and Bland-Altman analysis suggest that the segmentation results of the proposed model are in good agreement with the ground truth and the accuracy and clinical applicability for the automatic evaluation of LV function.

We also tested the improved ResUnet model on the LVQuan18 dataset, which originates from different equipment operated at multiple hospitals featuring a variety of resolutions. Images provided by LVQuan18 have ROIs with a size of 80 × 80 pixels that are extracted from 145 cases. Of the 145 cases, 130 are provided as the training set and the remaining 15 cases as the test set. The PGC, DM, and APD values of the endocardium/epicardium for the segmentation results on LVQuan18 are 100.00%/100.00%, 0.97/0.97, and 1.25 mm/1.37 mm, respectively. These results confirm the good adaptability of our proposed model to various data sets.

### 3.3. Comparisons

To verify the superiority of our proposed model in segmentation accuracy, we compared our results with Lu et al.'s image-driven method [5], Long et al.'s FCN model [14], Ngo et al.'s hybrid method of level set and deep learning [30], Kerfoot et al.'s residual U-Net method [23], Hu et al.'s combination of deep learning and dynamic programming method [9], and Wu et al.'s GAN model [25] on the same dataset of MICCAI 2009.  Table 2 lists the values of PGC, DM, and APD for each of these methods. The APD of the endocardium and epicardium with our model is 1.60 mm and 1.37 mm, respectively, which are better than the best APD values of 1.71 mm and 1.64 mm reported by others [25]. For the value of PGC, our proposed model is higher compared to the traditional algorithms, deep learning-based methods, and their combinations and 1.78% higher than the state-of-the-art model for the endocardium and 1.79% for the epicardium. The higher the PGC value, the more slices are involved in calculating the DM value. In this case, our endocardium and epicardium DM values of 0.93 and 0.96, respectively, are still higher than most other methods and the same as the most advanced method.

The *p* values of the Student's t-test for the distribution of the APD, PGC, and DM metrics for our model and Lu et al.'s, Kerfoot et al.'s, and Hu et al.'s algorithms are all less than 0.01. This result suggests that the segmentation results of our model are significantly different from those of these other algorithms. For Long et al.'s, Ngo et al.'s, and Wu et al.'s methods, due to the lack of relevant details, these algorithms cannot be reproduced, as we cannot give the *p* values between the results of our model and the others at this time.

All experimental results presented here show that our proposed model displays good segmentation performance and robustness, with superior metrics compared to other algorithms.

## 4. Discussion

**Figure 7:**
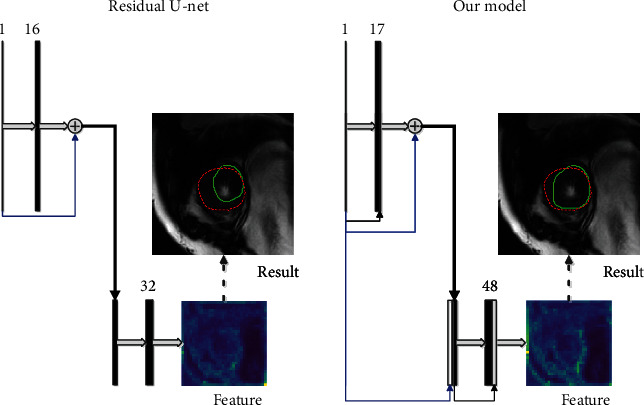
Comparison of units between the residual U-Net and our model. The solid green line represents the segmentation result, and the dotted red line represents the ground truth.

The quality of the convolution features impacts the performance of the CNN model. In our proposed model, the skip connection enhances the reuse of features and increases the sensitivity of the network to gradient changes. To compare the difference between our model and the residual U-Net [18], Figure 7 shows the units of the first down sampling in both models. As seen in this figure, the primary difference is that our model enhances the reuse of the input image of the model (blue arrow) and the initial feature of each step (black arrow) by introducing skip connections, which significantly enrich the information contained in the feature map. Also, in Figure 7, one of the real feature maps is selected from the convolution features after the first downsampling to observe its distribution. Here, the LV contour in our feature map is clearer compared to the residual U-Net. Correspondingly, the final segmentation result (solid green line) of our method is closer to the ground truth (dotted red line) compared to the residual U-Net, which is also consistent with the results in Table 3.

The effect of the depth-wise separable convolution on improving the efficiency of the model is verified by replacing the depth-wise separable convolution in the proposed “improved ResUnet” model with the ordinary convolution and recording the replaced model as the “ResUnet+skip connection.” Table 3 presents the segmentation results of the residual U-Net [23] (denoted as ResUnet), “ResUnet+skip connection,” and “improved ResUnet” (our model). As seen in Table 3, the ResUnet+skip connection enhances the reuse of features and improves the quality of the convolutional features compared to the original ResUnet. This practice improves the segmentation accuracy while increasing training time. Compared with the ResUnet+skip connection, the improved ResUnet introduces a depth-wise separable convolution, resulting in a lower cost of convolution and reduced training times for the endocardial segmentation from 354 s to 260 s/epoch and the epicardium from 200 s to 146 s/epoch. The PGC of the endocardium segmentation is also improved. Therefore, by introducing separable convolution into our model, we significantly reduce the training time and improve the efficiency of the model while ensuring sufficient segmentation accuracy.

**Figure 8:**
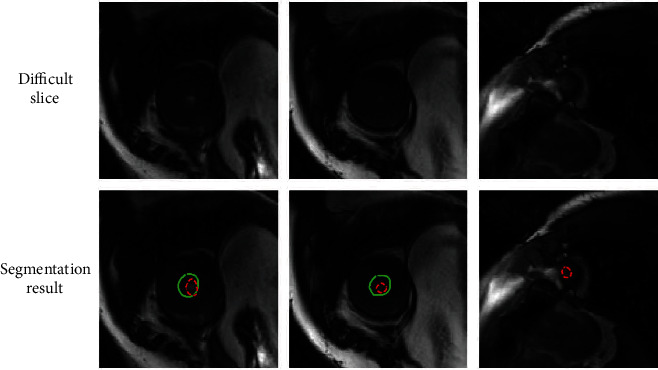
Segmentation results (solid green line) and ground truth (dotted red line) of three difficult slices.

Although our model achieved promising performance in LV segmentation, some issues remain that offer opportunities for further research, such as the segmentation of small and fuzzy LV from the apical slices in the ES phase. As illustrated in Figure 8, the top includes three difficult slices, and the bottom includes the corresponding segmentation results (solid green line) and ground truth (dotted red line). From left to right are the slices IM-0001-0127 in SC-HYP-37, IM-0001-0147 in SC-HYP-37, and IM-0001-0186 in SC-N-37. The APD for the left slice is 4.85 mm, which is considered a good segmentation, but the dice coefficient of 0.59 is relatively low. The APD for the middle slice is 6.25 mm, which is considered a poor segmentation. The right slice is not correctly segmented. All slices presented here are from the apical slices in the ES phase, with a very small blood pool area and endocardium that are not obvious. Improving the segmentation performance for such small targets will be addressed in future work.

## 5. Conclusion

An enhanced ResUnet model was proposed to improve the accuracy and efficiency of the endocardium and epicardium extraction process from cardiac MR images. Our contributions include introducing various skip connections to enhance feature reuse, improve the sensitivity of the model to gradient changes, and recover spatial information lost during down sampling for improving segmentation accuracy. Also, we introduced a separable convolution to further improve the model's efficiency. Our proposed method outperformed other baseline and state-of-the-art methods in terms of multiple assessment metrics. The comparison between our model and others (including the state-of-the-art method) suggests that the results of our model are highly consistent with the ground truth. Furthermore, our model achieves strong performance on the LVQuan18 dataset, which validates the promising adaptability of our model to various datasets.

## Figures and Tables

**Table 1:** Segmentation results on all 15 test cases.

Patient id	Endocardium			Epicardium		
	PGC (%)	DM	APD (mm)	PGC (%)	DM	APD (mm)
SC-HF-I-05	100.00	0.96	1.20	100.00	0.97	1.00
SC-HF-I-06	100.00	0.94	2.00	100.00	0.97	1.23
SC-HF-I-07	100.00	0.94	1.27	100.00	0.96	1.17
SC-HF-I-08	100.00	0.95	1.62	100.00	0.97	1.28
SC-HF-NI-07	100.00	0.92	2.25	100.00	0.97	1.40
SC-HF-NI-11	100.00	0.93	2.04	100.00	0.96	1.30
SC-HF-NI-31	100.00	0.93	1.51	100.00	0.96	1.16
SC-HF-NI-33	100.00	0.94	1.29	100.00	0.95	1.16
SC-HYP-06	100.00	0.91	1.25	100.00	0.95	1.39
SC-HYP-07	100.00	0.92	1.19	100.00	0.95	1.67
SC-HYP-08	100.00	0.91	1.61	100.00	0.96	1.43
SC-HYP-37	92.31	0.86	2.65	100.00	0.94	1.99
SC-N-05	100.00	0.91	1.44	100.00	0.95	1.36
SC-N-06	100.00	0.93	1.43	100.00	0.94	1.50
SC-N-07	94.44	0.93	1.32	100.00	0.94	1.54
MEAN	99.12	0.93	1.60	100.00	0.96	1.37
STD	2.29	0.02	0.42	0.00	0.01	0.23

PGC: percentage of good contours; DM: dice metric; APD: average perpendicular distance; MEAN: mean of all cases; STD: standard deviation; SC-HF-I: heart failure with ischemia; SC-HF-NI: heart failure without ischemia; SC-HYP: hypertrophy; and SC-N: normal.

**Table 2:** Comparison of segmentation results on MICCAI 2009.

Methods	Endocardium			Epicardium		
	PGC (%)	DM	APD (mm)	PGC (%)	DM	APD (mm)
Lu et al.^∗^ (2009)	77.63 (16.89)	0.89 (0.03)	2.07 (0.59)	85.68 (13.58)	0.94 (0.02)	1.91 (0.61)
Long et al. (2015)	95.23 (-)	0.80 (-)	1.95 (-)	95.62 (-)	0.85 (-)	2.14 (-)
Ngo et al. (2017)	95.91 (5.28)	0.88 (0.03)	2.34 (0.46)	94.65 (6.18)	0.93 (0.02)	2.08 (0.60)
Kerfoot et al.^∗^ (2018)	97.98 (4.56)	0.91 (0.03)	1.91 (0.47)	98.28 (4.07)	0.95 (0.02)	1.76 (0.32)
Hu et al.^∗^ (2019)	96.80 (7.0)	0.90 (0.03)	1.95 (0.48)	98.40 (6.5)	0.93 (0.02)	1.98 (0.53)
Wu et al. (2021)	97.34 (-)	0.93 (-)	1.71 (-)	98.21 (-)	0.96 (-)	1.64 (-)
Ours	99.12 (2.29)	0.93 (0.02)	1.60 (0.42)	100 (0.00)	0.96 (0.01)	1.37 (0.23)

PGC: percentage of good contours; DM: dice metric; APD: average perpendicular distance. Symbol ∗ denotes the *p* value for the distributions of this metric between our model and the corresponding method is lower than 0.01. Number format: mean value (standard deviation).

**Table 3:** Comparison of the effects of skip connection and separable convolution on MICCAI 2009.

Models	Endocardium			
	PGC (%)	DM	APD (mm)	TIME (s/epoch)
ResUnet [18]	97.98 (4.56)	0.91 (0.03)	1.91 (0.47)	290
ResUnet+skip connection	98.60 (2.63)	0.92 (0.03)	1.57 (0.42)	354
Improved ResUnet	99.12 (2.29)	0.93 (0.02)	1.60 (0.42)	260

Models	Epicardium			
	PGC (%)	DM	APD (mm)	TIME (s/epoch)
ResUnet [18]	98.28 (4.07)	0.95 (0.02)	1.76 (0.32)	152
ResUnet+skip connection	100 (0.00)	0.96 (0.01)	1.36 (0.24)	200
Improved ResUnet	100 (0.00)	0.96 (0.01)	1.37 (0.23)	146

PGC: percentage of good contours; DM: dice metric; APD: average perpendicular distance. Number format: mean value (standard deviation).

## Data Availability

The data that support the findings of this study are openly available in MICCAI 2009 at http://sourceforge.net/projects/cardiac-mr/ (reference number 27).
